# Simulation for training in sinus floor elevation: New surgical bench model

**DOI:** 10.4317/medoral.17701

**Published:** 2012-02-09

**Authors:** Juan Seoane, Javier López-Niño, Inmaculada Tomás, Antonio González-Mosquera, Juan Seoane-Romero, Pablo Varela-Centelles

**Affiliations:** 1 PhD, MD, DDS, Stomatology Department. School of Medicine and Dentistry. University of Santiago de Compostela. Spain; 2 DDS, Stomatology Department. School of Medicine and Dentistry. University of Santiago de Compostela. Spain; 3 PhD, DDS, Stomatology Department. School of Medicine and Dentistry. University of Santiago de Compostela. Spain

## Abstract

Objectives: to describe a bench model (workshop of abilities) for sinus floor elevation (SFE) training that simulates the surgical environment and to assess its effectiveness in terms of trainees’ perception. Study design: thirty-six randomly selected postgraduate students entered this cross-sectional pilot study and asked to fill in an anonymous, self-applied, 12-item questionnaire about a SFE workshop that included a study guide containing the workshop’s details, supervised practice on a simulated surgical environment, and assessment by means of specific check-lists. Results: Thirtiy-six fresh sheep heads were prepared to allow access to the buccal vestible. Using the facial tuber, third premolar and a 3D-CT study as landmarks for trepanation, the sinus membrane was lifted, the space filled with ceramic material and closed with a resorbable membrane. The participants agreed on their ability to perform SFE in a simulated situation (median score= 4.5; range 2-5) and felt capable to teach the technique to other clinicians or to undertake the procedure for a patient under supervision of an expert surgeon (median= 4; range 1-5 ). There were no differences on their perceived ability to undertake the technique on a model or on a real patient under supervision of an expert surgeon (p=0.36). Conclusions: Clinical abilities workshops for SFE teaching are an essential educational tool but supervised clinical practice should always precede autonomous SFE on real patients. Simulation procedures (workshop of abilities) are perceived by the partakers as useful for the surgical practice. However, more studies are needed to validate the procedure and to address cognitive and communication skills, that are clearly integral parts of surgical performance.

** Key words:**Sinus-floor elevation, animal model, dental education, dental implants, teaching, simulation.

## Introduction

Surgical training consists of developing cognitive, clinical, and technical skills -the latter traditionally acquired through mentoring (Halstedian training)-(1,2), being an ethical obligation to preserve the patient’s security and privacy during the learning process (3).

Mistakes are inherent to learning curves, but errors are not acceptable in surgical training: this makes simulation so attractive in the field of surgery, as it avoids the use of patients for skills practice and ensures that trainees have some practice before treating humans (4).

Different bench-top model settings based upon virtual simulations and workstations with synthetic, animal or cadaveric materials have proved an advantage for surgical training and improved the standard of education (2,5,6).

Sinus floor elevation (SFE) is one of the most widely used options for rehabilitating the posterior sector of the resorbed maxilla. In view of its important predictability (7,8) surgical skills are frequently learned in the operating room on live patients despite that existing bench-model training in laboratory settings may offer a valuable adjunct for learning basic surgical skills.

The information about sinus floor elevation procedures (modified Caldwell-Luc technique) in animal models is scarce (9-11). Neither the conceptual framework, nor the learning environment and the effectiveness to replicate this surgical situation have been described.

The aim of this study was to describe a new bench model (workshop of abilities) for SFE training that simulates the surgical environment and to assess its effectiveness in terms of trainees’ perception.

## Material and Methods

A cross-sectional pilot study was designed to describe a workshop on clinical abilities for SFE and to evaluate trainees’ perception about this bench-model. The thirty-six participants were selected by means of a table of random numbers from the postgraduate students of the University of Santiago de Compostela Oral Implantology Specialization Course that met the following inclusion criteria: previous surgical experience on implant insertion and lack of experience on SFE.

Sixteen participants were selected and asked to fill in an anonymous, self-applied, 12-item questionnaire.

This questionnaire was a modification of a previously used survey instrument (10) and was piloted among a convenient group to ensure practicability. The questions were broadly grouped into 2 sections: profiling questions (demographic and practice) and questions about the trainees’ perception on the usefulness of the workshop and also on their believed ability to undertake the technique on real patients. The answers had to be graded on a Likert-type scale (1 maximum disagreement – 5 maximum agreement).

-Workshop on SFE clinical abilities

Each participant received a study guide containing the workshop’s specific objectives, information on the technique (theoretical bases of the procedure, methodology and a list of typical errors and complications), together with anatomical details of the animal model, a list of the materials required and information on the assessment method. This study guide was read at the beginning of the session.

The workshop was developed at the Dental School’s abilities lab and the partakers were informed about the conditions of its use and safety regulations that were basically identical to those of a real surgical environment. The participants were divided into pairs and allocated to an adequate setting within the lab to individually undertake the procedure while the tutors provided intense external feedback to correct errors in the surgical technique.

The students were assessed by direct observation during the workshop (2 hours) by means of specific checklists that included topics related to intraoral approach of the maxillary sinus, incision and flap raising, lateral window opening, sinus membrane (integrity and elevation) and graft packing, along with membrane placement and flap repositioning and suture. Once the procedure was completed, students were allowed time for autonomous learning on the other maxillary sinus of the model.

-Statistical analysis

Data were entered on the SPSS 12.0 statistical package (SPSS Inc. Chicago, IL, USA), and the sample characterized by the variables of interest. Data distribution was defined by the mean and the median, as central trend statistics, and the standard deviation and the range as spread indicators. Quantitative variables were studied by means of non-parametric tests: the means were compared by the Friedman’s variance test (more than two means) and the Wilcoxon test (two means). The significance level chosen for all tests was p<0.05.

## Results

-Description of the surgical bench model 

Thirty-six fresh heads obtained from sheep younger than 12 months were used as a model for this ex-vivo study. The material was free from disease and transported from the slaughterhouse within 8 h post-mortem.

**Figure 1:**
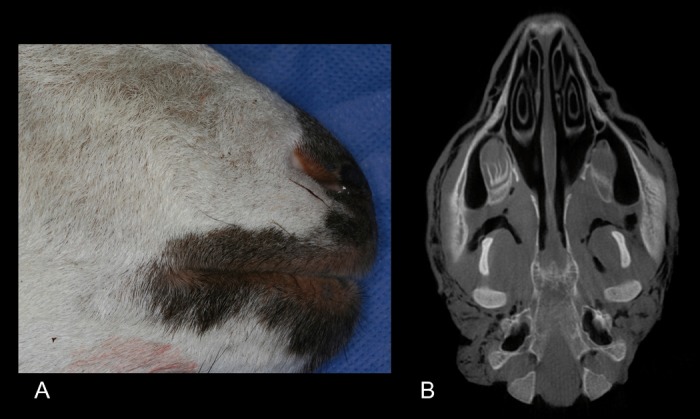
A) Sheep head, close view. B) i-CAT axial slice of the model.

**Figure 2:**
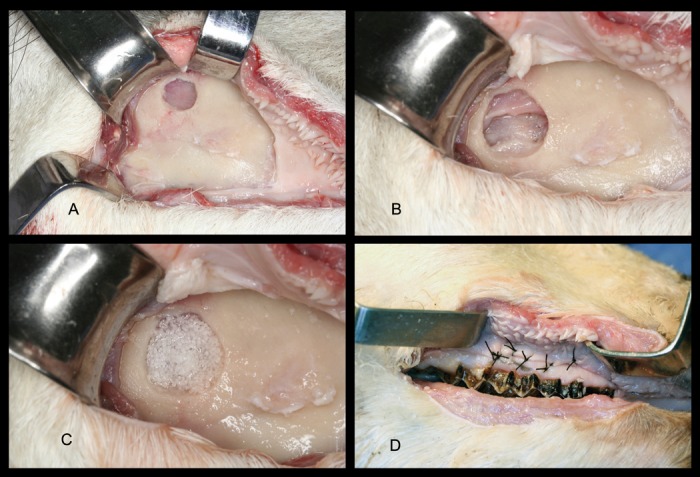
A) Full thickness flap reflection and sinus wall trepanation; B) Reflection and elevation of the Schneider’s membrane; C) Microparticulate graft in place; D) Flap repositioned and sutured.

All 36 specimens were studied by means of a cone beam CT (i-CAT, Imaging Sciences International, 1910 North Penn Toad, Hatfield PA, USA) and two incisions performed: a 3.5 cm skin incision caudally from the angle of the mouth and a 2 cm perpendicular dorsal incision to connect the former one, in order to ease access to the buccal vestibule (10). Each partaker used the facial tuber and third premolar as a landmark and the 3D study to facilitate the surgical approach to the maxillary sinus. The trepanation was undertaken with a round diamond bur (023; Komet, Lengo, Germany) mounted on a straight handpiece, and the sinus membrane lifted with an ad-hoc surgical kit (Mozo-Grau, Valladolid, Spain). The grafting material was specifically designed for simulation (Bone-Ceramic Simulator; Straumann SA, Madrid, Spain). Once the procedure was completed, a fibrin sponge membrane (Hemarcol, Dentsply spd; Bretonneux, France) was placed over the osseous fenestration and the flap repositioned and sutured (Figs. 1,2).

The working area was always framed by a fenestrated surgical drape.

-Students’ opinions on the SFE workshop

The mean age of the attendees was 33.1± 8.0 (41.7% males; n=15) that had been in practice for an average of 8.3 ±6.5 years. The participants said to be very interested in the workshop (mean score 4.6± 0.5; range 3 to 5).

The highest agreement rate was noted when asked about their ability to perform SFE in a simulated situation (median=4.5) and whether observation of other professional’s performance eased learning (median=5). On the other hand, most students considered themselves unable to undertake SFE on a real patient after completion of the workshop, but reported that they felt capable to teach the technique to other clinicians or to undertake the procedure for a patient under supervision of an expert surgeon (median = 4,  Table 1).

**Table 1:**
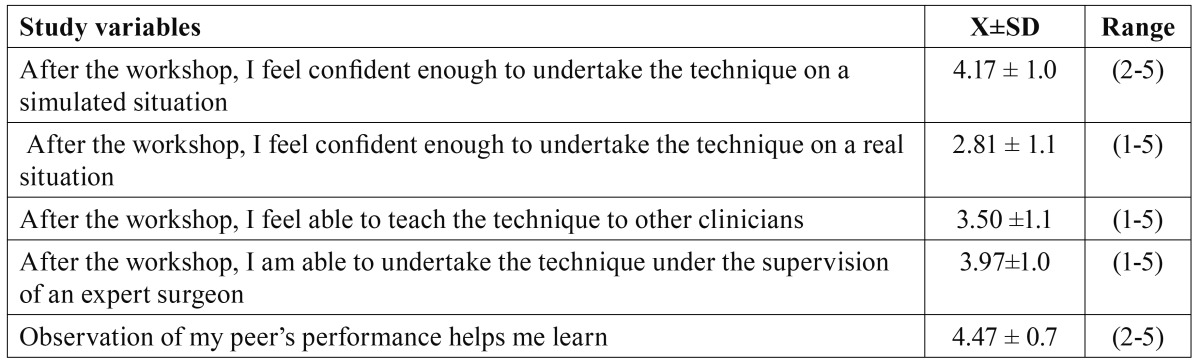
Partakers’ self-assessment of their capacities to undertake a SFE (Likert 1-5 scale).

Significant differences could be identified in terms of partaker’s self-perception of their ability to undertake SFE in different clinical situations (simulation vs. real patient vs. real patient under supervision) (p=0.0001), as the students felt more capable to perform SFE in a simulated situation than in a real one (p=0.0001), but there were no differences on their perceived ability to undertake the technique on a model or on a real patient under supervision of an expert surgeon (p=0.36).

## Discussion

-Conceptual framework

The classical method of “surgical perceptorship” as a method of learning manual operative skills, with orderly exposure to graduate clinical experience in the operating room under the close tutelage of dedicated senior surgeons (1,2), has been modified by the implementation of new learning environments -known as clinical abilities laboratories- due to practical, financial, ethical and theoretical advantages (3,12).

The simulated bench-model training provides the surgical educator with the opportunity to standardize the quality and quantity of hands-on practice, allowing for outcome errors and the use of feedback to correct them, as some form of feedback is required for motor learning to occur (2,13). Simulations in a simulated operation theatre permit the evaluation of both technical and non-technical abilities (pre-operative, communication, control of the situation, etc.) related to a specific surgical technique (14). In addition, simulation-based surgical training has also proved to reduce clinical errors and learning curves (2,13).

-Rationale for the bench-model 

Experimental studies in terms of sinus floor elevation procedures have been performed in different animal models including monkeys (15), rabbits (16), dogs (17), sheep (10), goats (18), mini-pigs (19) and domestic pigs (11). Animal selection for surgical training should take into account specimen availability, acceptability to the society, costs, and similarity between animal and human maxillary sinus (20,21). All these models have their strengths and weaknesses: economic and ethical reasons eliminate monkeys as an animal model for sinus floor elevation training, in spite of being the species most similar to humans (20); dogs show an osseous composition and a bone reorganization pattern close to human’s, but negative public perception of using companion animals for medical research limits its use (21); rabbits have also been widely used for research on regenerative procedures and grafting materials for SFE, but the technique for extraoral approach to the maxillary sinus hampers its use at clinical abilities workshops, where reliability and validity of the model depend on the realism of the simulation (2).

Swine demonstrate a good likeness with human bone (the most similar macrostructure), ex-vivo pig heads are cheap and easily available at slaughterhouses and their sinus morphology allows elevation heights of up to 10 mm ( but difficulties may be encountered in relation to their size and ease of handling for experimental use (21) and because intraoral surgical approach is difficult, with a very resistant cortical bone to trepanize (10) that limits the use of this model, as bone drilling performance is an important part of surgical expertise (22).

Sheep and goats meet most selection criteria, as being easily obtained, display minimal genetic variation, show large sinus cavities (larger than mature white pig), permit intraoral surgical approach, and are similar to human species when Schneider membrane and sinus osseous wall are considered (10). However, their heads (when elder than 12 months or after the eruption of a permanent incisor) are considered specific risk materials for spongiform encephalitis by the EU and specific regulations apply for the use of this animal models for surgical training.

The proposed bench-model is based upon the use of sheep younger than 12 months and thus free from the risk of prion transmission. This model not only meets the selection criteria for surgical training in SFE, but also the mechanical and physical properties of ovine bone are similar to humans’, as occurs with bone density from skeletally immature sheep (21,23).

-Workshop on clinical abilities for SFE

Although a number of papers have focused on developing animal models (simulators) for SFE, none of them has described or assessed the simulation process, that is, the development of an educational setting where learning the technique in a close-to-real, stress-free environment that does not depend on the availability of real patients and permits working either under supervision or on an independent basis (2). The acquisition of the knowledge, abilities, attitudes and values needed to undertake a task can not be achieved by an animal model only, isolated from a given context.

The attendees’ limited agreement about that workshop completion enables the student to autonomously undertake the procedure on a real patient is congruent with the fact that satisfactory performance on a simulator is by no means comparable to clinical competence. However, after workshop conclusion most partakers felt able to perform a SFE on a real patient under the supervision of an expert surgeon.

Generally speaking, the students have consider the workshop useful for achieving and improving their SFE abilities, which seems to support the idea of spreading this educational strategy. Team work and the opportunity to observe their pair’s performance were perceived as positive by the attendees for learning the technique. Similar feedback has been published for abilities workshops in other surgical specialties, like traumatology, gynecology and ENT (2).

Within the limitations inherent to these kind of studies, our results seem to suggest the convenience for including this clinical abilities workshop when teaching SFE, as it is an ancillary but essential educational tool and supervised clinical practice should always precede autonomous SFE on real patients.

More studies are needed to validate the procedure and to address cognitive and communication skills, that are clearly integral parts of surgical performance.
